# General practitioner residents and patients end-of life: involvement and consequences

**DOI:** 10.1186/s12910-022-00867-9

**Published:** 2022-12-03

**Authors:** Victoire Haardt, Amélie Cambriel, Sidonie Hubert, Marc Tran, Cédric Bruel, Francois Philippart, for the REQUIEM Study group

**Affiliations:** 1 Marie-Thérèse Medical Center, Paris, France; 2 Anesthesiology and Intensive Care Medicine Department, APHP-Tenon University Hospital, Paris, France; 3 Internal Medicine Unit, Groupe Hospitalier Paris Saint Joseph, Paris, France; 4 Medical and Surgical Intensive Care Unit, Groupe Hospitalier Paris Saint Joseph, Paris, France; 5 REQUIEM study group, Paris, France

**Keywords:** End-of-life, Patient autonomy, General practitioner, Patient care management, Junior physician, Psychological resilience, Clinical competences

## Abstract

**Background:**

The ageing of the population and the increased number of chronic diseases are associated with an increased frequency of end of life care in hospital settings. Residents rotating in hospital wards play a major part in their care, regardless of their specialty. General practitioner (GP) residents are confronted to such activities in hospital settings during their training. Our aim was to know how they feel about taking care of dying patients, as end-of-life care are very different from the clinical activity they are trained to.

**Methods:**

We surveyed all GP trainees of “Ile de France”. The survey was made of 41 questions regarding advanced directives divided in 7 sections about patients’ care, communication, mentoring and repercussion on personal life. The survey was done one time, during two pre-specified days.

**Results:**

525 residents (53.8%) accepted to fulfill the survey. 74.1% of the residents thought that palliative care could have been better. Possible ways of improvements were: a reduction of unreasonable obstinacy (or therapeutic overkill, two terms defined in French law as curative treatment without reasonable hope of efficiency) (59.6%), patient’s (210 answers, 40%) and relative’s communication (information of patients and relatives about the severity of the disease and risk of death) (199 answers 37.9%). Residents also reported a lack of knowledge regarding end-of-life care specific treatments (411 answers, 79.3%) and 298 (47.2%) wished for better mentoring. Those difficulties were associated with repercussion on their private life (353 answers, 67.2%), particularly with their close relatives (55.4%). Finally, 56.2% of trainees thought that a systematic psychologic follow up should be instituted for those working in “at risk” hospital settings.

**Conclusion:**

Self-perception management of dying patients by GP resident emphasize their lack of training and supervision. The feeling of suboptimal care is associated with consequences on personal life.

**Supplementary Information:**

The online version contains supplementary material available at 10.1186/s12910-022-00867-9.

## Background

The aging of the population and scientific improvement have led to the increase of chronic conditions [1, 2]. The progressive physiological alteration associated with long-term diseases may ultimately lead to death [3]. Thus, in the US, 70% of death is due to chronic illness [4]. On the same way, in France, two third of deaths are due to chronic illness and “could require end-of-life care” [5].

Most deaths, in particular those of patients who have chronic illnesses or require end-of-life care, take place in hospital settings [6–8]. In most cases, end of life care is provided in department that are not specialized in palliative care [9].

In this situation, care is not limited to pharmaceutical prescription. They also include delivering bad news such as a serious disease or a bad prognosis, and managing relatives before and after death. This kind of care, because of the confrontation to physical and moral distress, in particular in serious situations, is associated with a risk of psychological complications among caregivers [10, 11] but also senior physicians [12–15]. Having to take part in decisions of therapeutics’ withdrawal or withholding is associated with a greater risk [16–18].

In a vast number of department (for example oncology, neurology, internal medicine, …), prescriptions are made by residents and controlled by senior physicians. Although youth and inexperience are known factors for psychological and broadly moral distress [11, 15], most of the currently available works looking at psychological complications cares, especially of dying patients, has been done with experienced physician, in department often confronted to end of life care such as in oncology, hospice and trauma departments [15].

The aim of our work is to evaluate the psychological impact of end of life care in the GP residents’ population who is young and who is training for family medicine rather than palliative medicine.

## Methods

### Survey

The survey was designed by the REQUIEM group members and built according to professional guidelines [19, 20]. It was made of 41 questions divided in two major sections (Additional file 1: survey): knowledge regarding the French law on end of life care (2016-87- February 2nd, 2016; Claeys-Leonetti’s law) and end of life care management during their last previous rotation (6 months). The second section was divided in seven subsections looking at: participation in therapeutic withdrawal decisions, implementation of palliative care decision by the whole medical team, management of patients’ end of life care by the resident, repercussions of end-of-life care on the resident’s personal life, quality of resident’s supervision for end-of-life care patients, resident’s training regarding end-of-life care and professional goals. Of note, we use the term of “unreasonable obstinacy” (or previously “therapeutic overkill”), defined as all curative acts undertaken when they seem futile, without reasonable hope of effectiveness, in order to standardize resident response to the survey.

The survey was anonymous. Participation to the survey was orally offered to all general practitioners (GP) residents (“internes de médecine générale”) during a mandatory interview (a global meeting, including all residents, for their choice of next rotation), between March 29th and April 5th 2016. This specific time and place was planned to allow the best response rate, as all residents were presents. The survey was filled out on a voluntary basis. Residents who did not want to participate in the study only needed to give back the survey unfilled to the investigator (VH).

### Population

In France medical training is separated in two periods: six years of general formation (“externat”) followed by 3 to 6 years of residency depending on the specialty (“internat”). For general practitioners, residency last 3 years. In those three years GP residents complete six 6-month rotations either in hospital wards or in GP’s practice.

We surveyed all GP residents training in Paris and its suburbs (Ile de France). The survey was looking at their last rotation, in any hospital ward. They were all met during a specific and mandatory gathering (administrative meeting to choose their next rotation) by the first author (VH).

### Ethics

The questionnaire was anonymous and filled out on a voluntary basis. An oral informed consent to participate was obtained from all of the participants in the study. Residents who declined to answer had to make it clear by handing out a blank survey to the investigator (VH). Empty surveys were collected to determine the number of participants involved.

The study is in compliance with the Helsinki Declaration. According to French legislation this survey was a non-interventional study and therefore did not require submission to an ethics committee per se. The survey was, however, approved by our hospital ethics board, who did not request any change and gave us their agreement. We did not need to obtain formal consent from the respondents. A filled-out survey was considered non-opposition to the study. Participants were informed that the data obtained would be anonymously analyzed and published in a scientific journal.

### Statistical analysis

Data were expressed as mean and standard deviations or, if appropriate, median and confidence intervals as a function of response disparity.

Comparison of variables was analyzed by an exact Fisher test or Chi^2^. Differences were considered as statistically significant for a p value of less than 5%. Statistical analysis was done on Statistica®.For ease of reading, the most statistically significant results are indicated in bold in the tables

## Results

**Fig. 1:**
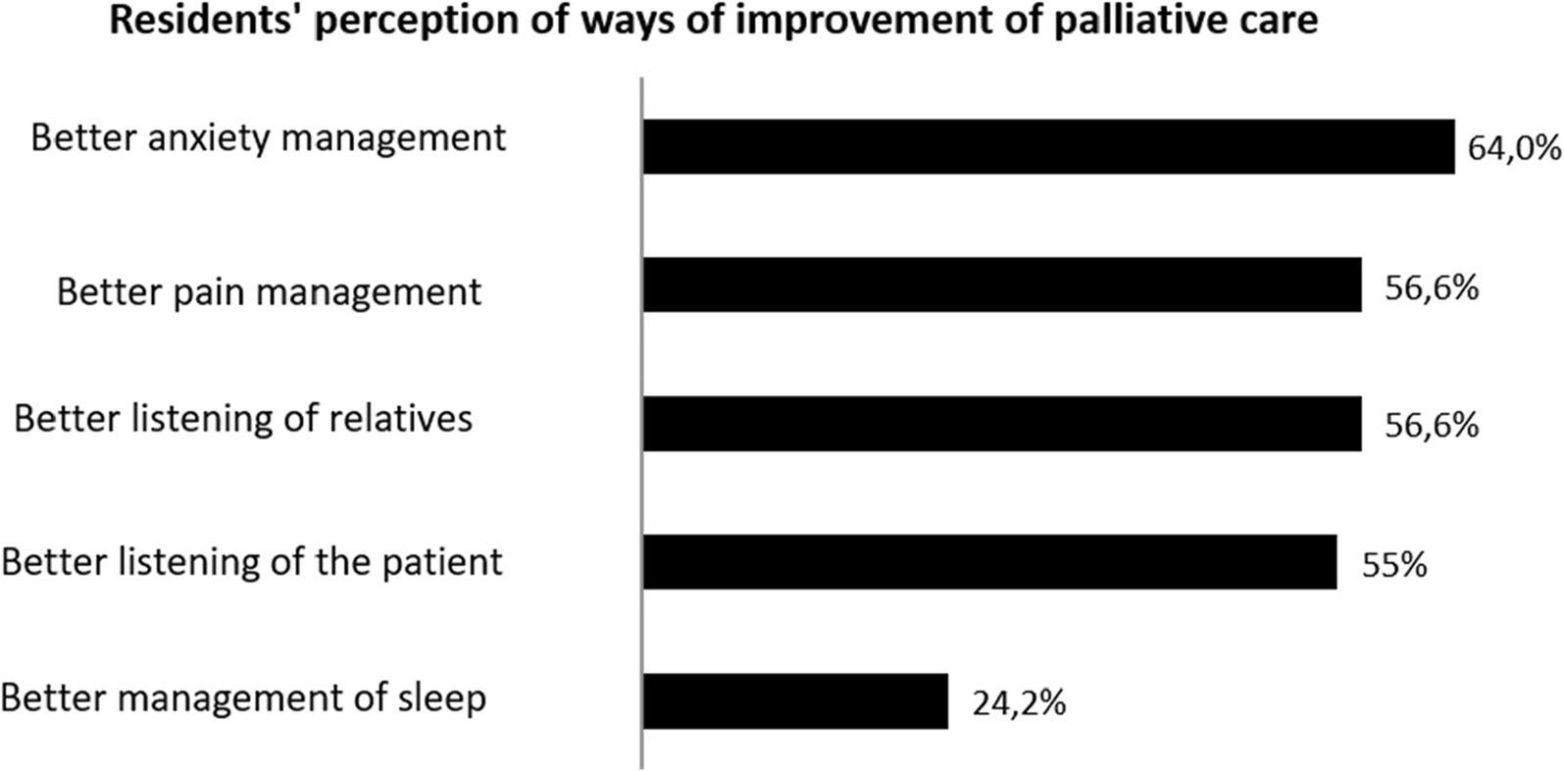
Residents’ perception of ways of improvement of palliative care

**Fig. 2:**
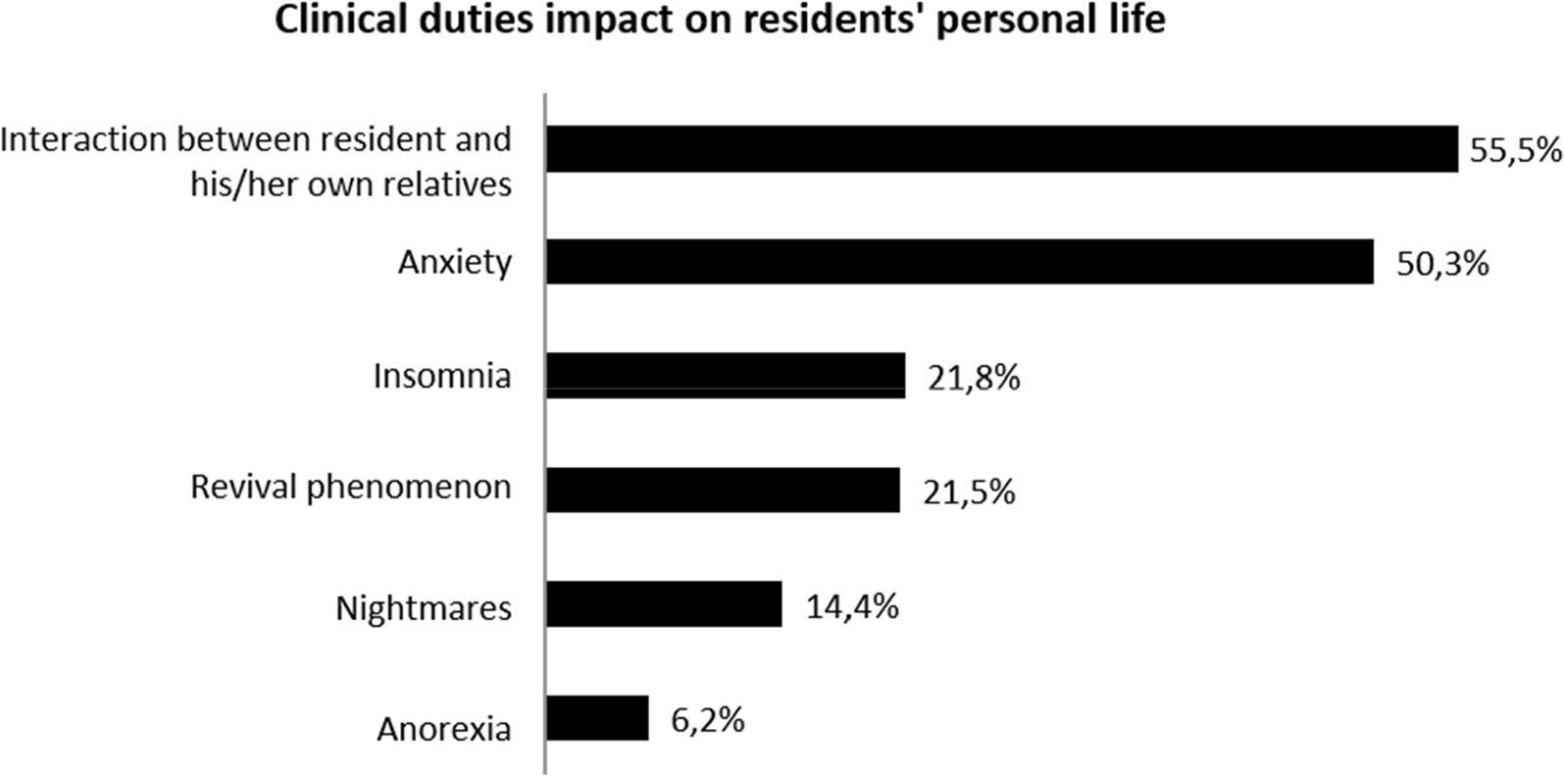
Parameters associated with Clinical duties impact on residents' personal life

**Fig. 3:**
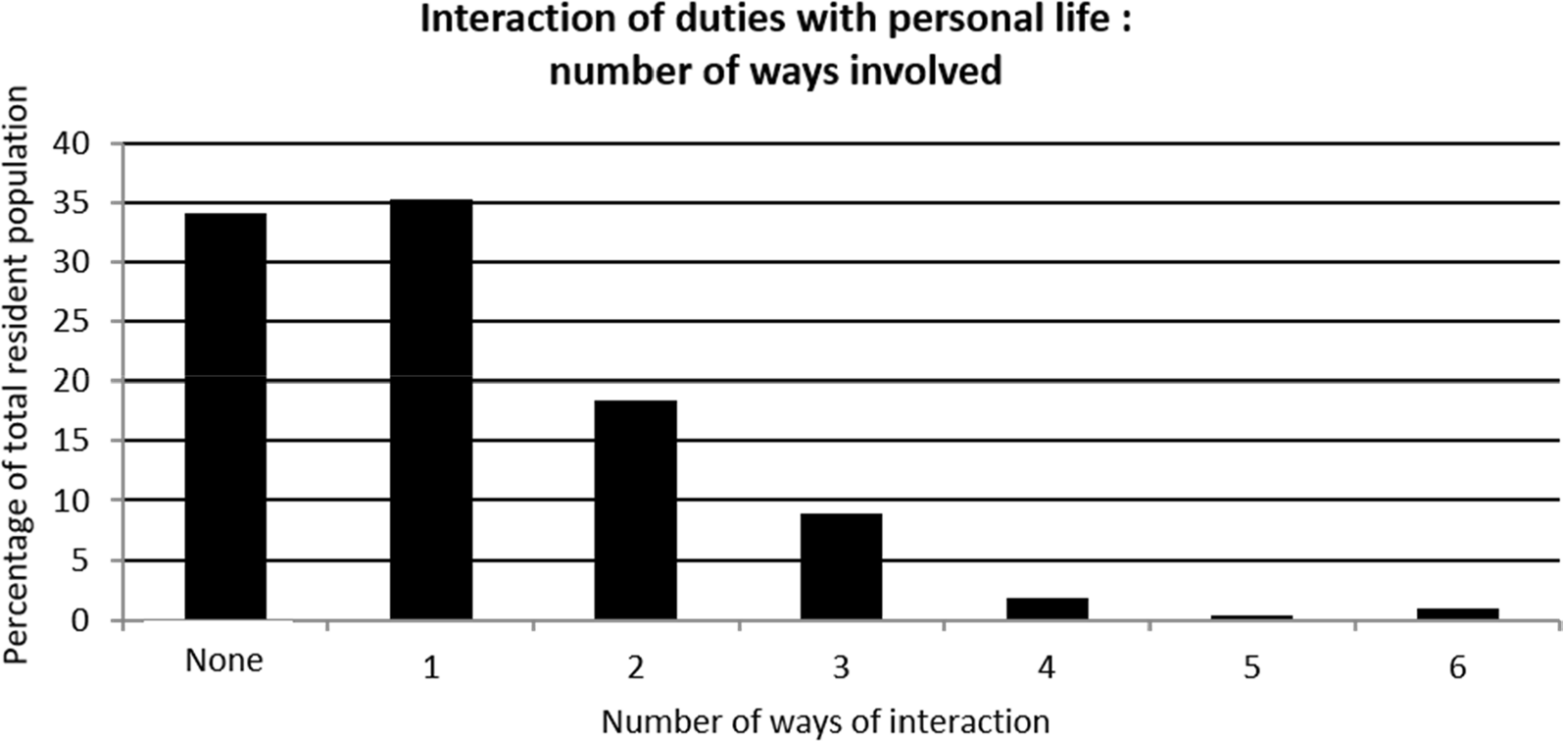
Number of ways of interactions with personal life

**Fig. 4:**
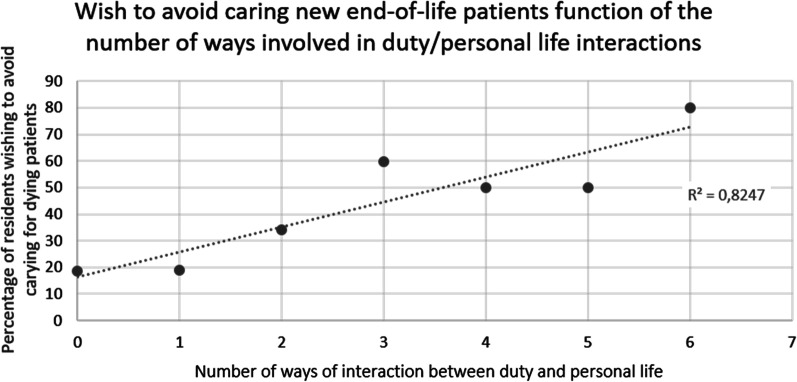
Correlation between the Wish to avoid caring new end-of-life patients and the number of ways involved in duty/personal life interactions

1. Population
  The survey was handed out to all GP residents working in Paris and its suburbs (“Île-de-France”) (n = 1172). About them, 196 (16.7%) did not fulfilled the inclusion criteria (previous 6 months’ rotation in GP office and not in hospital ward). Among the remaining 976 potential responders, 525 (53.8%) accepted to answer and fulfilled the survey. The remaining residents (n = 451, *i.e.* 46.2% of potential responders and 38.4% of the whole residents’ population) refused to fill out the survey after being informed.
2. Patients’ end-of-life care during their current rotation
  354 (67.4%) residents were satisfied with the quality of end of life care during their rotation. However, 315 (60%) thought that palliative care should have been implemented earlier. Moreover, 74.1% of them thought that the end-of-life care could have been improved. Possible axis for improvement were both somatic (pain) (64%) and anxiety (56,6%) management and psychological support for the patient and his relatives (See Fig. 1).
  The period of time immediately preceding death was particularly difficult since 239 residents (45.4%) considered that patients’ physical suffering was inefficiently treated. Finally, 58.5% of the GP residents felt that their own psychological distress was not correctly taken care of.
3. Parameters associated with the perception of end-of-life care quality
  Four Types of parameters are associated with resident’s evaluation of end-of-life care quality: end-of-life care implementation, quality of patient’s complaints’ support, residents’ perception of their management by senior physicians and personal difficulties regarding end-of-life care. Details are in Table 1.
4. Difficulties met by residents while caring for dying patients
  - Perception of unreasonable obstinacy (lack of communication between physicians)
    313 residents (59.6%) consider having witnessed unreasonable obstinacy in the care of their hospitalized patients. They considered it was obstinacy because of the severity of the clinical picture (65.9%) (example: a patient with multimetastatic cancer and severe malnutrition, with a loss of autonomy—clinical frailty scale: 7 -, who has been offered and umpteenth line of chemotherapy) and because of the severity of the prognosis short-term (61.7%) (example of testimony during the survey: “the end-of-life was obvious for the whole team except the attending physician, and the patient died without comfort treatment”). For 15.1% of residents, obstinacy was revealed by the refusal to follow a previously stated therapeutics’ limitation.
  - Communication with senior physicians
    Among residents who felt having witnessed unreasonable obstinacy, only 167 (53.4%) felt free to express their disagreement.
  - Communication with patients
    210 (40%) participants disclosed having difficulties to talk about death with their dying patients. Likewise, 199 (37.9%) residents have difficulties talking about these things with patients’ relatives. Both difficulties are associated in 63.2% of cases.
    As expected the wish to stop caring for dying patients in the future and communications difficulties are linked (cf. Table 2). Conversely, wishing to have an end-of-life activity in the future is associated with a lesser risk of communication difficulties. (cf. Table 2).
  - Therapeutic difficulties
    Only 114 (21.7%) residents say having no difficulties using and adapting analgesic and sedative drugs used in end-of-life care. Also, 46.9% of the residents admit their fear to shorten the life of these patients because of the use of sedative and analgesic treatments. Moreover, 63.4% (n = 246) of them considered that this fear to shorten life may have limited the adaptation of end-of-life care treatments. In the same fashion, fear to shorten life was associated with the feeling of a lack of anxiety management for dying patients (p = 0,007).
  - Need for stronger supervision
    In this work, 227 (52.8%) residents were satisfied with the supervision form senior staff members for the end-of-life care.
    Among residents considering that supervision could improve (n = 248), 59.7% of them wished for more assistance in the therapeutic aspect of end-of-life care.
    The quality of supervision is associated in our work to the perceived quality of end-of-life care. However, it is not possible to determine the causality (cf. satisfaction towards end-of-life care and Table 3).
5. Distress in end-of-life care management during the current rotation
  - Distress related to the perception of unreasonable obstinacy
    Among residents who felt they were witnessing unreasonable obstinacy (n = 313), 64.6% were hurt by the situation. This distress was associated with the feeling to be unable to express their disagreement on the course of treatment in 71.7% of cases. Distress was also increased when feeling that the end-of-life care was insufficient (cf Table 4). Conversely, the perceived quality of supervision what associated with a lesser risk of distress.
  - Repercussion on personal life
    The need to care of dying patients in the context of their hospital rotation is associated with negative impact on their personal life for 67.2% (n = 353) of the surveyed residents.
    Among them, 55.4% (n = 195) felt that their professional work impacted their relationship with their close relatives (example of testimony during the survey: “I *was so concerned about my dying patient that my friend's problems seemed trivial*”, “*they told me I gradually lost my patience during the period of hospital work and they were waiting for it to end*”). 50.4% (n = 178) felt they were more anxious after these end-of-life care (“*I felt that I no longer knew how to prescribe, including for everyday treatments*”; “*everything I was told became a source of anxiety, without me knowing why, and often without a clear idea of what was making me anxious*”); 21.5% (n = 76) had reliving phenomenon; 20.8% (n = 74) suffered from insomnia after these events (“*I woke up every night for no reason at 2:35 a.m. exactly and couldn't go back to sleep because of my patient whose condition I was constantly worried about. The more I was tired, the more the situation was eating me up*”). The details of these elements are in Fig. 2 and Table 5.
    Residents having felt an interaction with their personal life are more prone to wish to avoid end-of-life care in the future (30.9% versus 17.4%; p = 0,001).
    The number of ways of interactions with personal life differs depending on the residents (cf. Figure 3). In about one third of resident, only one type of the personal life parameters (interaction with relatives, anxiety, insomnia, revivals, nightmares, anorexia) was affected by their palliative care activity. The impact can include two or more of these parameters and even include the six ways of interaction (1%; n = 5). The proportion of residents wishing to avoid being responsible for dying patients depends on the number of personal life’s settings previously touched by this situation (R^2^: 0.8247) (Fig. 4).
    We observed a link between the impact on private life and the perceived quality of care. Residents who feel their clinical duties impact their personal life often thought the end of life care of their patient was not sufficient. They also have more difficulties in their communications with their patients (resident hurt by the perception of unreasonable obstinacy; perception of difficulties talking about death with their patient; details in Table 5).
    As expected, residents having felt an impact of end-of-life care on their personal life wished more for a systematic psychological support in *at risk* departments. (61.5% *vs* 45.3%; p = 0.0004).
6. Impact of difficulties met: desire to avoid caring for dying patients.
  Factors associated with the desire not to care for any new dying patients were: the fear to shorten life with end-of-life care treatments (analgesic and anxiolytics) (p = 0.0002), communication difficulties with the patient and their relatives (p = 5.32.10^–6^ and p = 0.0004 respectively), impact on personal life (p = 0.001).
  Conversely, the quality of end-of-life care (p = 1.21.10^–5^), residents’ supervision by seniors (p = 0.0002), and desire to have an end-of-life care activity in the future (p = 0.05) were factors associated with the absence of avoidance of end-of-life care patients. Details are in Table°6.
7. Potential interest of a psychological support for residents

**Table 1:** Parameters associated with resident satisfaction about quality of patients’ end-of-life care

	Yes (n = 354) (%)	No (n = 171) (%)	p
*Delay in implementation of palliative care*			
Satisfaction about quality of patients’ end-of-life care			
Palliative care should have been implemented earlier	50**.**3	80**.**1	**6.2.10**^**–11**^
Perception of an unreasonable obstinacy	57**.**3	64**.**3	0**.**12
The patient took part in therapeutic intensity	61	57**.**9	0**.**49
Quality of palliative care could have been improved	65**.**3	92**.**4	**2.9.10**^**–11**^
Management of patients complaints			
Perception of an insufficient consideration of patient pain	40**.**7	55**.**6	**0.001**
Perception of an insufficient consideration of patient psychological frailty	56**.**8	62	0**.**25
Perception of quality of supervision form senior staff members			
Satisfied by the quality of their supervision	64**.**7	28**.**1	**3.37.10**^**–15**^
Perception of an ability to express their disagreement on the course of treatment	46**.**7	55**.**9	0**.**52
Distress in end-of-life care management			
Hurt by a perception of unreasonable obstinacy	60**.**9	70**.**9	0**.**077
No difficulties using and adapting analgesic and sedative drugs	23**.**2	18**.**7	0**.**24
Difficulties /avoiding talking about death with patient	40**.**1	52	**0.009**
Difficulties /avoiding talking about death with patient’s relatives	33**.**9	46**.**2	**0.006**
Desire to avoid caring for dying patients	20**.**6	38**.**6	**1.21 × 10**^**–5**^
Clinical duties impact their personal life	65**.**3	71**.**3	0**.**16
Wish to have a professional orientation in palliative care management	20**.**6	14	0**.**068

The most relevant statistically significant results are indicated in bold

**Table 2:** Factors associated with difficulties/avoiding talking about death

	Yes (n = 231) (%)	No (n = 294) (%)	p
*Factors associated with difficulties/avoiding talking about death with patient*			
Wish to have a professional orientation in palliative care management	13**.**4	22**.**4	0**.**008
Resident satisfied with the quality of end of life care during their rotation	61**.**5	72**.**1	0**.**009
No difficulties using and adapting analgesic and sedative drugs	19**.**5	17**.**9	0**.**64
Difficulties /avoiding talking about death with patient’s relatives	63**.**2	18	**3.3 × 10**^**–26**^
Desire to avoid caring for dying patients	36**.**4	18**.**7	**5.32 × 10**^**–6**^

	Yes (n = 199)	No (n = 326)	p
*Factors associated with difficulties/avoiding talking about death with patient*			
Wish to have a professional orientation in palliative care management	12**.**1	22**.**1	**0.004**
Resident satisfied with the quality of end of life care during their rotation	60**.**3	71**.**8	**0.006**
No difficulties using and adapting analgesic and sedative drugs	20**.**6	22**.**4	0**.**63
Difficulties /avoiding talking about death with their patients	73**.**4	18	**3.3 × 10**^**–26**^
Desire to avoid caring for dying patients	35**.**2	21**.**2	**0.0004**

The most relevant statistically significant results are indicated in bold

**Table 3:** Perception of the quality of their supervision by senior staff members

	Yes (n = 354) (%)	No (n = 171) (%)	p
*Perception of the quality of their supervision by senior staff members*			
Satisfied by the quality of their supervision	64.7	28.1	**3**.**37 × 10**^**–15**^
Felt free to express their disagreement	46.7	55.9	0.52

The most relevant statistically significant results are indicated in bold

**Table 4:** Suffering due to a Perception of unreasonable obstinacy

	Yes (n = 201) (%)	No (n = 111) (%	p
*Suffering due to a Perception of unreasonable obstinacy*			
Resident satisfied with the quality of end of life care during their rotation	61	71.2	0.072
Palliative care should have been implemented earlier	75.1	55	**0**.**0002**
Perception of an insufficient consideration of patient pain	51.7	36	**0**.**007**
Perception of an insufficient consideration of patient psychological frailty	59.7	55	0.41
Felt free to express their disagreement about a perceived unreasonable obstinacy	48.3	62.7	**0**.**01**
No difficulties using and adapting analgesic and sedative drugs	23.4	26.1	0.59
Difficulties /avoiding talking about death with their patients	68.5	40.4	0.21
Difficulties /avoiding talking about death with patient’s relatives	39.6	32.6	0.27
Clinical duties impact their personal life	77.1	57.7	**0**.**0003**
Desire to avoid caring for dying patients	29.4	19.8	0.065
Satisfied by the quality of their supervision	42.3	63.1	**0**.**0004**
Wish to have a professional orientation in palliative care management	21.4	13.5	0.08

The most relevant statistically significant results are indicated in bold

**Table 5:** Clinical duties impact on residents’ personal life

	**Yes (n = 353)**	**No (n = 172)**	**p**
Resident satisfied with the quality of end of life care during their rotation	65.4	71.5	0.16
Hurt by the perception of unreasonable obstinacy	70.8	49.5	**0**.**0003**
No difficulties using and adapting analgesic and sedative drugs	21.2	22.7	0.71
fear to shorten life by adaptation of end-of-life care treatments	47	46.5	0.91
Difficulties/avoiding talking about death with patient	47.3	37.2	**0**.**02**
Difficulties/avoiding talking about death with patient’s relatives	39.1	35.5	0.42
Satisfied by the quality of their supervision	48.2	62.2	**0**.**002**
Desire to avoid caring for dying patients	30.9	17.4	**0**.**001**
Wish to have a professional orientation in palliative care management	19.3	16.9	0.50
Would like a systematic psychological support in at risk departments	61.5	45.3	**0**.**0004**

The most relevant statistically significant results are indicated in bold

**Table 6:** factors associated with the desire to avoid caring for dying patients

	Yes (n = 139)	No (n = 386)	p
Quality of palliative care could have been improved	78.4	72.5	0.17
Perception of unreasonable obstinacy	58	60.2	0.65
Hurt by the perception of unreasonable obstinacy	72.8	61.5	0.065
Resident satisfied with the quality of end of life care during their rotation	52.5	72.8	**1**.**21 × 10**^**–5**^
The patient took part in therapeutic intensity	62.6	59.1	0.46
No difficulties using and adapting analgesic and sedative drugs	20.1	22.3	0.60
fear to shorten life by adaptation of end-of-life care treatments	60.4	42	**0**.**0002**
Difficulties /avoiding talking about death with patient	60.4	38.1	**5**.**32 × 10**^**–6**^
Difficulties /avoiding talking about death with patient’s relatives	50.4	33.4	**0**.**0004**
Clinical duties impact their personal life	78.4	63.2	**0**.**001**
Satisfied by the quality of their supervision	39.6	57.5	**0**.**0002**
Wish to have a professional orientation in palliative care management	12.9	20.5	**0**.**05**
Would like a of a systematic psychological support in at risk departments	68.3	51.8	**0**.**0007**

The most relevant statistically significant results are indicated in bold

Among residents who filled out the survey, 56.2% thought that a systematic psychological support should be implemented in *at risk* departments to decrease the risk of extraprofessional impact of the care of severe patients. Departments that were considered at risk were oncology, hematology, critical care or geriatrics. Factors associated with the demand for a psychological follow up are: distress related to the perception of unreasonable obstinacy (p = 0,028), interaction of those care with the resident’s personal life (p = 0.00004), and the desire to avoid new end-of-life care (p = 0.0007). Conversely, residents who were satisfied with their supervision did not feel as strongly the need for a psychological support (p = 0.005).

## Discussion

Our work has pointed out that GP residents having finished at least one rotation in a hospital ward have many difficulties regarding the end-of-life care. The first obstacle standing is the implementation of comfort care itself because of a lack of mastery in the use of pain and anxiety medicine in the specific context of end-of-life care. The difficulty they have in communicating about death also plays an important role in the trauma felt by our population. Also, the feeling of giving insufficient care because of those difficulties has a negative impact on the residents’ personal life. All of these factors lead the most affected residents to avoid future contact with palliative care.

End of life care is an important part of medical training. Although family medicine is usually about caring for patients with less severe pathology, family doctors often have to care for patients wishing to die at home [21]. A specific training during medical school has been implemented and strengthened in the last decades, but our results underline the persistent difficulties met by physicians. Our study blends in the current work about young physicians’ preoccupations regarding end-of-life care [9], even though the population is younger and working in a different place than the one they are training to work in the future. Despite those differences, results are similar [9], which underlines that difficulties in apprehending end-of-life care depends very little on the professional orientation of young physicians.

The quality of end-of-life care is the main preoccupation of GP residents. Imperfections experienced by our population were associated with a lack of ease of sedatives and anxiolytic treatments use as well as communicating with the patient and their relatives. Such feeling of unpreparedness toward those situations has been widely reported and may concern as far as 80% of young physicians [22]. The lack of theoretical training, in particular regarding pharmacological end of life care therapeutics, is often reported [22–27]. Some work from the beginning of the year 2000 pointed out this lack of training amongst general practitioners [28]. Although the medical training has been widely modified since, it seems to still be true.

Patients with fatal disease often ask for the possibility to discuss end of life care with their physician [29]. However, young physicians [9, 30, 31] as well as residents [30] are faced with difficulties in communicating in this situation. In the same fashion, residents find it difficult to determine the right time for implementing palliative care as well as the practical management of patients’ complaints. These difficulties have also been reported by internal medicine residents [23] who are more used to these end-of-life care situations, including during their critical care rotations [24].

Relations with relatives may need particular attention. Indeed, the severity of the illness or disagreements, regarding the orientation and intensity of care, may be cause for tensions between relatives and physicians and might be difficult to accept in the particular context of end of life care [32]. Also, satisfaction of grieving families depends on the quality of medical attention both in end of life care decisions and organization as well as in the post death support [33].

Lack of practice seems to be the main cause for communication difficulties. The absence of specific training in end of life communication is associated with physician discomfort and unease feelings [34–36]. Consequently, it is also a risk factor for burn-out [15]. Bedside training is often offered, but associated with a poor psychological tolerance [27]. High fidelity scenarios as well as seminars, while confronting residents to pragmatic situation without the psychological burden, might improve residents’ future competence [23, 37]. Distance training has also proven to be efficient to improve young physicians’ communication skills [31]. This improvement of end of life care management’s skills is associated with a diminution of anxiety in these situations [38]. Conversely, lack of trust in ones skills and communication, in particular in breaking bad news, are at the center of health care worker’s psychological distress [12, 15].

In our work, all of these elements are associated with a psychological burden on more than two third of replying GP residents. Similar results were obtained in different populations of young and inexperienced physicians, also leading to psychological distress, similar to post-traumatic disorders [9]. Patient’s loss may lead to psychological distress because of a sense of guilt, or even burn-out. [39]. This distress is mostly due to death events [40, 41] and uncertainty around end-of-life care [38]. Death is often accountable for guilt feelings and sometimes even sense of failure in physicians populations [27]. However, most of the publications about the effects of end-of-life care or care in severe situations are looking at experienced physicians in specialized departments [15].

As expected, supervision plays an import role in GP residents’ mental health and satisfaction during end-of-life care [26, 42, 43]. Lack of involvement, management and discussions lead to a situation of discomfort when faced to end-of-life care [26, 44]. In our work, help from senior physicians is associated with perceived good quality of care by the resident and with a lower risk of personal burden related to end-of-life care. This confirms previous observations in different populations [40]. Senior management remains, however, sometimes insufficient for young physicians [25, 26, 44, 45].

Supervision and adequate management medication use at end-of-life and decision-making process, must, however, not lead to the exclusion of residents during this care. Their exclusion could alter their training during residency [26] as experience depends on seniors ‘observation [46].

On a broader perspective, training is essential to prevent psychological consequences of medical activities. The quality of end-of-care training is correlated to patient’s end of life quality [47].

Our work has some limitations. Since the survey was filled out on a voluntary basis, the answers are not reflecting the mind of all residents. However, since the survey was offered to all residents orally, the absence of answer was not due to a lack of solicitation. Absence of participation was always due to participation’s refusal. Observed results confirm that participants were the most interested in end-of-life care (18% of replier wish to keep an end-of-life care activity in the future). Although they wish to care for end-of-life patients, perception of a lack of skills and psychological burden of this care on personal life are often reported, thus confirming their importance. Also, we only surveyed “Île-de-France” residents rather than all French residents. Residents from “Île-de-France” however represent almost a third of all French residents and are from all regions of France which allows an important representativeness of all residents. Finally, since answers were declarative, we cannot have any certainty about the reality of difficulties met and psychological impact. However, this limitation is common in this kind of work and links between psychological distress and perceived difficulties have been previously reported in similar situations with different physicians’ populations.

Our work allows a broad evaluation of the difficulties and consequences met by GP residents when they have to care for end-of-life patients in hospital settings. It also underlines the necessity to continue and strengthen their management during their hospital rotations.

## Conclusion

End-of-life care is often provided during hospital rotations. Although many improvements in the medical formation have been made, residents often report insufficient competence regarding specific end-of-life care therapeutics and in patients and relatives’ communication. These gaps in their training is associated with a negative impact on residents’ personal life. It may lead to the desire to avoid end-of-life care in the future.

## Supplementary Information

**Additional file 1:** Survey regarding GP residents’ perception of end-of-life care in hospital’s wards in “Ile de france” (/ Paris area).

## Data Availability

Data are available on motivated demand to the corresponding author (FP).

## Abbreviations

GP: General practitioner
REQUIEM: Research/Reflexion on End of Life Support Quality in Everyday Medical Practice
US: United States of America

## References

[1] Mathers CD, Loncar D (2006). Projections of global mortality and burden of disease from 2002 to 2030. PLoS Med.

[2] Gomes B, Calanzani N, Gysels M, Hall S, Higginson IJ (2013). Heterogeneity and changes in preferences for dying at home: a systematic review. BMC Palliat Care.

[3] Sharp T, Moran E, Kuhn I, Barclay S (2013). Do the elderly have a voice? Advance care planning discussions with frail and older individuals: a systematic literature review and narrative synthesis. Br J Gen Pract.

[4] Teitelbaum HS, Travis LD, Heilig DL, Neslund SE, Menze AK, Baker CD (2013). The epidemiology of hospice and palliative care. Dis Mon.

[5] Observatoire national de la fin de vie, éditeur. Observatoire national de la fin de vie. Rapport 2011: Fin de vie: un premier état des lieux. [Internet]. 2011. Disponible sur: http://www.ladocumentationfrancaise.fr/var/storage/rapports-publics/124000093.pdf

[6] Poulalhon C, Rotelli-Bihet L, Moine S, Fagot-Campagna A, Aubry R, Tuppin P (2018). Use of hospital palliative care according to the place of death and disease one year before death in 2013: a French national observational study. BMC Palliat Care.

[7] Higginson IJ, Daveson BA, Morrison RS, Yi D, Meier D, Smith M (2017). Social and clinical determinants of preferences and their achievement at the end of life: prospective cohort study of older adults receiving palliative care in three countries. BMC Geriatr.

[8] Pivodic L, Pardon K, Morin L, Addington-Hall J, Miccinesi G, Cardenas-Turanzas M (2016). Place of death in the population dying from diseases indicative of palliative care need: a cross-national population-level study in 14 countries. J Epidemiol Commun Health.

[9] Bharmal A, Morgan T, Kuhn I, Wee B, Barclay S (2019). Palliative and end-of-life care and junior doctors’: a systematic review and narrative synthesis. BMJ Support Palliat Care.

[10] Piers RD, Azoulay E, Ricou B, Dekeyser Ganz F, Decruyenaere J, Max A (2011). Perceptions of appropriateness of care among European and Israeli intensive care unit nurses and physicians. JAMA.

[11] St Ledger U, Reid J, Begley A, Dodek P, McAuley DF, Prior L (2021). Moral distress in end-of-life decisions: a qualitative study of intensive care physicians. J Crit Care.

[12] Pereira SM, Fonseca AM, Carvalho AS (2011). Burnout in palliative care: a systematic review. Nurs Ethics.

[13] Philip J, Gold M, Schwarz M, Komesaroff P (2007). Anger in palliative care: a clinical approach. Intern Med J.

[14] Ramirez AJ, Graham J, Richards MA, Cull A, Gregory WM (1996). Mental health of hospital consultants: the effects of stress and satisfaction at work. Lancet.

[15] Kearney MK, Weininger RB, Vachon MLS, Harrison RL, Mount BM (2009). Self-care of physicians caring for patients at the end of life: « being connected... a key to my survival ». JAMA.

[16] Aubry R. Etat des lieux du développement des soins palliatifs en France en 2010. Rapport à M. le président de la république et M. le premier ministre. [Internet]. Disponible sur: http://social-sante.gouv.fr/IMG/pdf/Rapport_Etat_des_lieux_du_developpement_des_soins_palliatifs_en_France_en_2010.pdf

[17] ANAES, SFAP. L’accompagnement des personnes en fin de vie et de leurs proches. Conférence de consensus [Internet]. 2004. Disponible sur: http://www.has-sante.fr/portail/upload/docs/application/pdf/Accompagnement_court.pdf

[18] ANESM : Agence nationale de l’évauation et de la qualité des établissements et services sociaux et médico-sociaux. Lettre de cadrage. Recommandations de bonnes pratiques professionnelles: accompagner la fin de vie des personnes âgées au domicile ou en établissement médico-social. 2015.

[19] de Singly F (2016). Le questionnaire.

[20] Gerard MFJ. Conduite d’enquête par questionnaire. 1re éd. Editions du robot furieux - Frederic Gerard; 2015: 122.

[21] Kjellstadli C, Allore H, Husebo BS, Flo E, Sandvik H, Hunskaar S (2020). General practitioners’ provision of end-of-life care and associations with dying at home: a registry-based longitudinal study. Fam Pract.

[22] Weil J, Gold M, McIver S, Rotstein L, Philip J (2012). Australian resident doctors want more palliative medicine education: a survey of attitudes and perceived needs. Intern Med J.

[23] Kawaguchi S, Mirza R, Nissim R, Ridley J (2017). Internal medicine residents’ beliefs, attitudes, and experiences relating to palliative care: a qualitative study. Am J Hosp Palliat Care.

[24] Centofanti J, Swinton M, Dionne J, Barefah A, Boyle A, Woods A (2016). Resident reflections on end-of-life education: a mixed-methods study of the 3 wishes project. BMJ Open.

[25] Bowden J, Dempsey K, Boyd K, Fallon M, Murray SA (2013). Are newly qualified doctors prepared to provide supportive and end-of-life care? A survey of foundation year 1 doctors and consultants. J R Coll Phys Edinb.

[26] Gibbins J, McCoubrie R, Forbes K (2011). Why are newly qualified doctors unprepared to care for patients at the end of life?. Med Educ.

[27] Schroder C, Heyland D, Jiang X, Rocker G, Dodek P (2009). Canadian researchers at the end of life network. Educating medical residents in end-of-life care: insights from a multicenter survey. J Palliat Med.

[28] Barclay S, Wyatt P, Shore S, Finlay I, Grande G, Todd C (2003). Caring for the dying: how well prepared are general practitioners? A questionnaire study in Wales. Palliat Med.

[29] Steinhauser KE, Christakis NA, Clipp EC, McNeilly M, McIntyre L, Tulsky JA (2000). Factors considered important at the end of life by patients, family, physicians, and other care providers. JAMA.

[30] Tait GR, Hodges BD (2013). Residents learning from a narrative experience with dying patients: a qualitative study. Adv Health Sci Educ Theory Pract.

[31] Clayton JM, Butow PN, Waters A, Laidsaar-Powell RC, O’Brien A, Boyle F (2013). Evaluation of a novel individualised communication-skills training intervention to improve doctors’ confidence and skills in end-of-life communication. Palliat Med.

[32] Cook D, Rocker G (2014). Dying with dignity in the intensive care unit. N Engl J Med.

[33] Hinkle LJ, Bosslet GT, Torke AM (2015). Factors associated with family satisfaction with end-of-life care in the ICU: a systematic review. Chest.

[34] Ury WA, Berkman CS, Weber CM, Pignotti MG, Leipzig RM (2003). Assessing medical students’ training in end-of-life communication: a survey of interns at one urban teaching hospital. Acad Med.

[35] Dunn A, Litrivis E (2011). Aligning patient preferences and patient care at the end of life. J Gen Intern Med.

[36] Herzler M, Franze T, Dietze F, Asadullah K (2000). Dealing with the issue « care of the dying » in medical education—results of a survey of 592 European physicians. Med Educ.

[37] Downar J, Knickle K, Granton JT, Hawryluck L (2012). Using standardized family members to teach communication skills and ethical principles to critical care trainees. Crit Care Med.

[38] Brennan N, Corrigan O, Allard J, Archer J, Barnes R, Bleakley A (2010). The transition from medical student to junior doctor: today’s experiences of tomorrow’s doctors. Med Educ.

[39] St Ledger U, Begley A, Reid J, Prior L, McAuley D, Blackwood B (2013). Moral distress in end-of-life care in the intensive care unit. J Adv Nurs.

[40] Paice E, Rutter H, Wetherell M, Winder B, McManus IC (2002). Stressful incidents, stress and coping strategies in the pre-registration house officer year. Med Educ.

[41] Moores TS, Castle KL, Shaw KL, Stockton MR, Bennett MI (2007). « Memorable patient deaths »: reactions of hospital doctors and their need for support. Med Educ.

[42] Harrington AW, Oliveira KD, Lui FY, Maerz LL (2020). Resident education in end-of-life communication and management: assessing comfort level to enhance competence and confidence. J Surg Educ.

[43] Smith-Han K, Martyn H, Barrett A, Nicholson H (2016). That’s not what you expect to do as a doctor, you know, you don’t expect your patients to die." Death as a learning experience for undergraduate medical students. BMC Med Educ.

[44] Linklater GT (2010). Educational needs of foundation doctors caring for dying patients. J R Coll Phys Edinb.

[45] Vivekananda-Schmidt P, Vernon B (2014). FY1 doctors’ ethicolegal challenges in their first year of clinical practice: an interview study. J Med Ethics.

[46] Wheatley-Price P, Massey C, Panzarella T, Shepherd FA, Mikhael J (2010). Resident preparedness in discussing prognosis in patients with advanced lung cancer. Support Care Cancer.

[47] Gillan PC, van der Riet PJ, Jeong S (2014). End of life care education, past and present: a review of the literature. Nurse Educ Today.

