# Supplementary material for: General practitioner residents and patients end-of life: involvement and consequences
Source: BMC Med Ethics. 2022 Dec 3;23:123. doi: 10.1186/s12910-022-00867-9 (PMC9719227; doi:10.1186/s12910-022-00867-9)
Supplement: Supplementary file 1 — Additional file 1: Survey regarding GP residents’ perception of end-of-life care in hospital’s wards in “Ile de france” (/ Paris area). [file 12910_2022_867_MOESM1_ESM.docx]

Survey regarding GP residents’ perception of end-of-life care in hospital’s wards in “ Ile de france” (/ Paris area)

The aim of our work is to collect your perception regarding these deaths.

Would you like to specialize in a field where end-of-life care will be required?

Yes

No

During your winter rotation:

1. Inside the hospital department :
2. End-of-life care organization

Were you satisfied with your patient’s end of life care?

Yes

No

Did you find your ward was unable to initiate palliative care with end-of-life care outreach team?

Yes

No

Did you feel that end-of-life care could have been initiated earlier?

Yes

No

If so: in your opinion was it related to the medical team’s feeling of failure when stopping curative treatments?

Yes

No

Did you feel palliative care could be of better quality?

Yes

No

If so: In your opinion what could have been improved?

Better pain management

Better anxiety management

Better sleep management

Better patient’s listening skills

Better patient’s relatives’ listening skills

1. Patients and relatives implications

Did patient participate in their therapeutic limitation?

Yes

No

Was their opinion gathered regarding the place they wanted to die in

Yes

No

In the same fashion, was relatives’ opinion gathered?

Yes

No

1. Pain, anxiety and sedative treatments

Are you completely comfortable with adapting dosages of pain sedatives and anxiety medicine for end-of-life care patients?

Yes

No

Did you feel that your patients were insufficiently physically relieved when dying?

Yes

No

Did you feel that your patients were insufficiently morally relieved when dying?

Yes

No

Did you fear to quicken the death of an end-of-life care patient when adapting his pain/sedatives’ treatments?

Yes

No

If so, did this fear limit your therapeutic adaptation?

Yes

No

1. Regarding you perception of unreasonable obstinacy :

Did you witness unreasonable obstinacy in explorations or treatment started in a patient who was in the terminal phase of his chronic disease:

Yes

No

If so did you feel free to express your disagreement?

Yes

No

Did you suffer from this situation / was this situation painful for you?

Yes

No

In your opinion it was unreasonable obstinacy because:

Your patient’s clinical state was too far advanced

Prognosis was already considered as very severe

Previous decision of therapeutic limitation had been decided

Patient had already refused the offered treatment or exploration

1. Regarding your perception of active therapeutics’ limitation

Have you ever avoided talking about death with a patient?

Yes

No

Have you ever avoided talking about a patient’s death with his relatives?

Yes

No

If so, can this avoidance been due to an emotional fatigue related with your professional environment?

Yes

No

Have you ever had the feeling that you were emotionless when facing these end-of-life care situations?

Yes

No

Have you ever felt that end-of-life care was interfering with your personal life?

Yes

No

If so on what aspects of your life?

Loss of appetite

Insomnia

Nightmares

Reviviscence phenomenon

Anxiety

Interections with your relatives

After those situations did you wish to avoid caring for other end-of-life care patients?

Yes

No

Do you think a systematic psychological follow-up would be necessary for residents working in “at risk” departments (oncology, hematology, geriatrics…)?

Yes

No

B. Regarding your management:

In your opinion, you management by your superior for end-of life care patients was sufficient?

Yes

No

If not: What aspects could have been improved?

Patient’s therapeutic management

Patient’s psychological management

Hierarchy’s listening skills of their residents

Supervised resident’s participation in therapeutic’s limitation decisions

1. Regarding your formation :

In your opinion is there a direct link between a good end-of-life care training and optimal care for dying patients?

In total agreement

In agreement

Rather in agreement

Rather not in agreement

In complete disagreement

Don’t know

In your opinion, a good training in end-of-life care for residents would improve:

The quality of the therapeutic aspect of end-of-life care

The quality of the psychological part of end-of-life care

The global quality of end-of-life care

Do you think that the training you received during medical school (“externat”) was sufficient to be prepared to care for end-of-life care patients and families?

Yes

No

Do you think it is advisable to improve end-of-life care training during residency?

Yes

No

Do you think it is necessary to have more theoretical lecture regarding end-of-life care during your residency?

Yes

No

Do you think it is necessary to have more practical training in end of life care during your residency? (Role plays, discussion about end-of-life care…)

Yes

No

We thank you for your participation in this study
